# Effects of dietary iron deficiency or overload on bone: Dietary details matter

**DOI:** 10.1016/j.bone.2024.117092

**Published:** 2024-04-02

**Authors:** Ulrike Baschant, Brie K. Fuqua, Maria Ledesma-Colunga, Christopher D. Vulpe, Stela McLachlan, Lorenz C. Hofbauer, Aldons J. Lusis, Martina Rauner

**Affiliations:** a Department of Medicine III & Center for Healthy Aging, Technische Universität Dresden, Germany; b Department of Physiological Sciences, University of Florida Center for Environmental and Human Toxicology, University of Florida, Gainesville, FL 32611, USA; c Usher Institute, The University of Edinburgh, Edinburgh, UK; d Department of Medicine, Division of Cardiology, David Geffen School of Medicine, University of California, Los Angeles, CA 90095, USA

**Keywords:** iron overload, iron deficiency, iron diet, Bone, Mouse strains

## Abstract

**Purpose:**

Bone is susceptible to fluctuations in iron homeostasis, as both iron deficiency and overload are linked to poor bone strength in humans. In mice, however, inconsistent results have been reported, likely due to different diet setups or genetic backgrounds. Here, we assessed the effect of different high and low iron diets on bone in six inbred mouse strains (C57BL/6J, A/J, BALB/cJ, AKR/J, C3H/HeJ, and DBA/2J).

**Methods:**

Mice received a high (20,000 ppm) or low-iron diet (~10 ppm) after weaning for 6–8 weeks. For C57BL/6J males, we used two dietary setups with similar amounts of iron, yet different nutritional compositions that were either richer (“TUD study”) or poorer (“UCLA study”) in minerals and vitamins. After sacrifice, liver, blood and bone parameters as well as bone turnover markers in the serum were analyzed.

**Results:**

Almost all mice on the UCLA study high iron diet had a significant decrease of cortical and trabecular bone mass accompanied by high bone resorption. Iron deficiency did not change bone microarchitecture or turnover in C57BL/6J, A/J, and DBA/2J mice, but increased trabecular bone mass in BALB/cJ, C3H/HeJ and AKR/J mice. In contrast to the UCLA study, male C57BL/6J mice in the TUD study did not display any changes in trabecular bone mass or turnover on high or low iron diet. However, cortical bone parameters were also decreased in TUD mice on the high iron diet.

**Conclusion:**

Thus, these data show that cortical bone is more susceptible to iron overload than trabecular bone and highlight the importance of a nutrient-rich diet to potentially mitigate the negative effects of iron overload on bone.

## 1. Introduction

Iron is essential for several physiological processes. As such, well-balanced iron levels are indispensable for health, with iron deficiency causing various health problems such as anemia, weakness and shortness of breath, and iron overload leading to severe organ damage if left untreated [1,2]. Body iron homeostasis is primarily controlled by absorption of dietary iron in the upper small bowel, as no regulated excretory pathway exists in mammals. Hepcidin, a peptide hormone produced in the liver, is the central regulator of systemic iron homeostasis and its expression is regulated by a host of factors including iron concentrations in the liver. It inhibits iron release from cells such as enterocytes and macrophages by binding to the iron exporter ferroportin and prompting its degradation [3] [4]. Properly managing tissue and cellular iron levels is critical as free iron can generate oxygen free-radicals, causing damage to biological molecules (e.g. DNA, lipids) [5].

Bone is amongst other tissues particularly sensitive to fluctuations in systemic iron levels [6] and requires balanced iron levels for proper function. At the cellular level, osteoblasts and osteoclasts both require iron for proper differentiation and function. Studies in vitro with iron chelating agents showed that iron deficiency dampens osteoclast differentiation [7] and promotes osteoblast function and differentiation [8]. In conditions of iron overload, the generation of reactive oxygen species increases osteoclast generation and activity, thereby leading to bone loss [9]. Osteoblasts, on the other hand, do not tolerate excessive iron levels well. High iron levels inhibit osteoblast differentiation and mineralization in vitro and in mouse models of genetic iron overload [8,10]. In line with this, patients with genetically caused iron overload or diseases with iron-loading anemias such as thalassemia, sickle cell anemia, or myelodysplastic syndromes show an increased presence of osteoporosis [11–13]. In patients with HFE-hereditary hemochromatosis in particular, an increased incidence of osteoporosis of up to 40 % and an increased fracture risk dependent on the extent of iron overload was reported [11]. Moreover, the increased risk for osteoporosis was also identified in a large independent cohort of patients with HFE mutations from the UK Biobank [5]. Finally, serum ferritin, a commonly measured clinical parameter positively associated with body iron stores, was negatively correlated with bone mineral density in a study of females aged 12–49, while higher serum ferritin levels were associated with osteoporosis and fractures in women aged over 45 [14–16].

Despite the large number of studies reporting detrimental effects of iron overload on bone, the underlying mechanisms remain incompletely understood. Various animal models of iron overload have been used to gain deeper insights into the pathogenesis. However, bone loss is not consistently observed in rodent models of genetically-caused hemochromatosis, such as the Hfe-knockout mouse, or in mice receiving iron injections or overload via the dietary route [10,17–21]. Potential reasons for the different outcomes include the different genetic backgrounds of the mice used, differing iron exposure in the studies, and/or the different ages used. Thus, the aim of our study was to evaluate the effects of dietary iron overload and iron deficiency on bone microarchitecture in male and female mice with a consistent and comparable dietary alterations in iron levels. Thereby, we focused on six different inbred strains to investigate in detail the impact of genetic background when studying iron effects on bone. Moreover, we compared the bone outcomes of high and low iron in two different dietary setups – either richer or poorer in minerals and vitamins – in C57BL/6J male mice.

## 2. Materials and methods

### 2.1. Mouse cohorts

Mouse studies were performed in two cohorts: the “UCLA” cohort at the University of California, Los Angeles, and the “TUD” cohort at the Technical University of Dresden, Germany. Mouse breeding and experimental procedures for the UCLA cohort were approved by the Institutional Care and Use Committee at University of California, Los Angeles (ARC#1992–169), while the TUD cohort study was approved by the Institutional Animal Care and Use Committee of the TU Dresden and the Landesdirektion Sachsen (TVV 20/2020). Both studies were performed according to the ARRIVE guidelines [22]. Mice were maintained in groups of up to 4 animals per cage and were kept in a 12-h light/dark cycle at room temperature in filter top cages with cardboard houses as enrichment. Mice were randomly assigned to treatment groups, and the subsequent analyses were performed in a blinded fashion.

For the TUD cohort (Dresden), 18 four week-old male C57BL/6JRj mice (six per group) were purchased from Janvier Labs (Saint Berthevin, France) and then fed one of three purified diets starting at 4 weeks of age: an iron free diet (<10 ppm iron, E15510), a standard diet (168 ppm iron, i.e. diet E15510 supplemented with 168 ppm iron, S8624-E710), or an iron rich diet (20,000 ppm, i.e. diet E15510 supplemented with 2 % carbonyl iron, S8624-E712, with diets all from ssniff Spezialdiäten, Germany) for 8 weeks. At 12 weeks of age, mice were sacrificed for bone and blood analyses.

The mice in the UCLA cohort are the subset of six classical inbred strains (A/J (RRID:IMSR_JAX:000646), AKR/J (RRID: IMSR_JAX:000648), BALB/cJ (RRID:IMSR_JAX:000651), C3H/HeJ (RRID:IMSR_JAX:000659), C57BL/6J (RRID:IMSR_JAX:000664), and DBA/2J (RRID:IMSR_JAX:000671) from a previously reported study (“Study 2” in [23]). In total, 180 male and female mice in the UCLA cohort were obtained from the Jackson Laboratory (Bar Harbor, Maine) and transferred to one of three diets at 4 weeks of age: a low iron diet (12 ppm iron, cat #115111; Dyets, Bethlehem, PA), a high iron diet (20,000 ppm iron as carbonyl iron, cat# 115122; Dyets), or an iron sufficient standard diet (50 ppm iron, cat# 515005; Dyets). There were six mice per sex, strain, and diet group, with the exception of only 5 females from the AKR/J and C57BL/6J strains in the high iron diet group. Only male mice were put on the low iron diet. At 10 weeks of age, mice were sacrificed for bone and blood analyses. The detailed compositions of all study diets are described in Suppl. Table 1.

### 2.2. Tissue collection

For the TUD cohort, mice were euthanized under isoflurane anesthesia and blood was taken by cardiac puncture and serum was collected after 10 min centrifugation at 400*g* in a non-fasted, non-perfused state. For the UCLA cohort, mice were fasted for four hours and then were euthanized under isoflurane anesthesia by exsanguination and cardiac perfusion with PBS (to flush tissues of blood), followed by cervical dislocation as previously described [23]. For both studies, tissues were excised, snap frozen in liquid nitrogen, and then stored at −80 °C.

### 2.3. Blood count analysis

For the TUD cohort, blood was transferred into EDTA blood collection tubes and diluted 1:3 with 0.9 % sodium chloride for blood count analysis. The blood parameters (RBC, HCT, HGB, MCV, MCH, MCHC, PLT and WBC) were determined using the Sysmex XN-1000. For the UCLA cohort, blood was analyzed as previously described [23]. The blood count data for the UCLA study male mice fed the iron sufficient standard and high iron diets has been previously reported in another publication where bone was not studied ([24], “pilot study” results).

### 2.4. Iron measurements

For the TUD cohort, non-heme iron content in the liver was measured using the bathophenanthroline colorimetric method as previously described [20]. In brief, 100 mg liver tissue was dried for 3 days at 37 °C and afterwards the samples were digested, followed by the reaction with 0.01 % bathophenanthrolinedisulfonic acid. Values were recorded spectrophotometrically at 535/540 nm. Non-heme iron content is reported as μg iron/g dry tissue weight.

For the UCLA cohort, total liver iron was measured in male mice by inductively coupled plasma-atomic emission spectroscopy (ICP-AES) as previously described [23] and in female mice by inductively coupled plasma-mass spectrometry (ICP-MS) as previously described [24]. The liver iron data for the UCLA study male mice fed the iron sufficient standard and high iron diets has been previously reported in another publication where bone was not studied ([24], “pilot study” results).

### 2.5. Assessment of bone microarchitecture

A vivaCT40 (ScancoMedical, Switzerland) was used for bone microarchitecture analysis of the femora of both cohorts ex vivo. The bones were imaged at an isotropic voxel-size of 10.5 μm with Xray energy of 70 kVp/114 μA and 200 ms of integration time. Following standard protocols from Scanco Medical, trabecular (Tb.) bone parameters including bone volume/total volume (BV/TV), trabecular number (Tb.N), trabecular separation (Tb.Sp) and thickness (Tb.Th) were determined based on calculations including 100 scan slices starting 20 slices below the growth plate. The diaphyseal region was located halfway between the femoral head and distal condyles and consisted of 150 slices. μCT parameters were reported according to international guidelines [25]. Bones from the UCLA cohort were sent frozen on dry ice to Dresden, and all bone analyses were completed there.

### 2.6. Serum analysis of bone turnover markers

Plasma samples from the UCLA cohort were shipped frozen on dry ice to Dresden for analysis. Serum concentrations of pro-collagen type I amino-terminal propeptide (P1NP) and C-terminal telopeptide (CTX) were quantified using enzyme-linked immunosorbent assays (Immundiagnostik, Bensheim, Germany), according to the manufacturer’s protocols.

### 2.7. Statistical analysis

Data are presented as mean ± standard deviation (SD). A two-sided Student’s *t*-test was used when comparing two groups and one-way analysis of variance (ANOVA) was performed when comparing more than two groups. Calculations were performed using GraphPad Prism 8 (GraphPad Software Inc., San Diego, CA, USA). *P*-values <0.05 were considered statistically significant.

## 3. Results

### 3.1. Results from the UCLA study

#### 3.1.1. Iron and blood parameters

To investigate the impact of dietary iron overload or deficiency on bone homeostasis in six different mouse inbred strains, we used bone samples from a previously published study that collected but did not examine bone (UCLA study, [23,24]. Therein, male and female mice of the C57BL/6J, A/J, BALB/cJ, AKR/J, C3H/HeJ, and DBA/2J strains were fed a low iron (12 ppm iron), a high iron (20,000 ppm iron), or an iron sufficient standard diet (50 ppm iron) starting from weaning for 6 weeks. Mice fed the low iron diet exhibited reduced liver iron concentrations, while mice receiving the high iron diet had elevated liver iron concentrations compared to mice on the iron sufficient standard diet (Table 1 and Supplemental File 1). Across all strains, C57BL/6J male mice on the high iron diet showed the greatest fold change (~30 fold) in iron levels when compared to the standard diet as previously reported [24], and in general with the exception of AKR/J mice, female mice accumulated less iron in the liver on the high iron diet than males (Table 1, and Supplemental File 1). Moreover, the low iron diet in males led to a drop in hemoglobin, hematocrit, and mean cell volume in most strains, but overall had little effect on red blood cell count. In contrast, in male and female AKR/J, C57BL/6J and DBA/2J mice, the high iron diet led to a significant drop in red blood cell count, hemoglobin, and hematocrit, and an increase in mean cell volume (Table 1 and Supplemental File 1).

#### 3.1.2. Effects of iron overload on bone

**Fig. 1.**
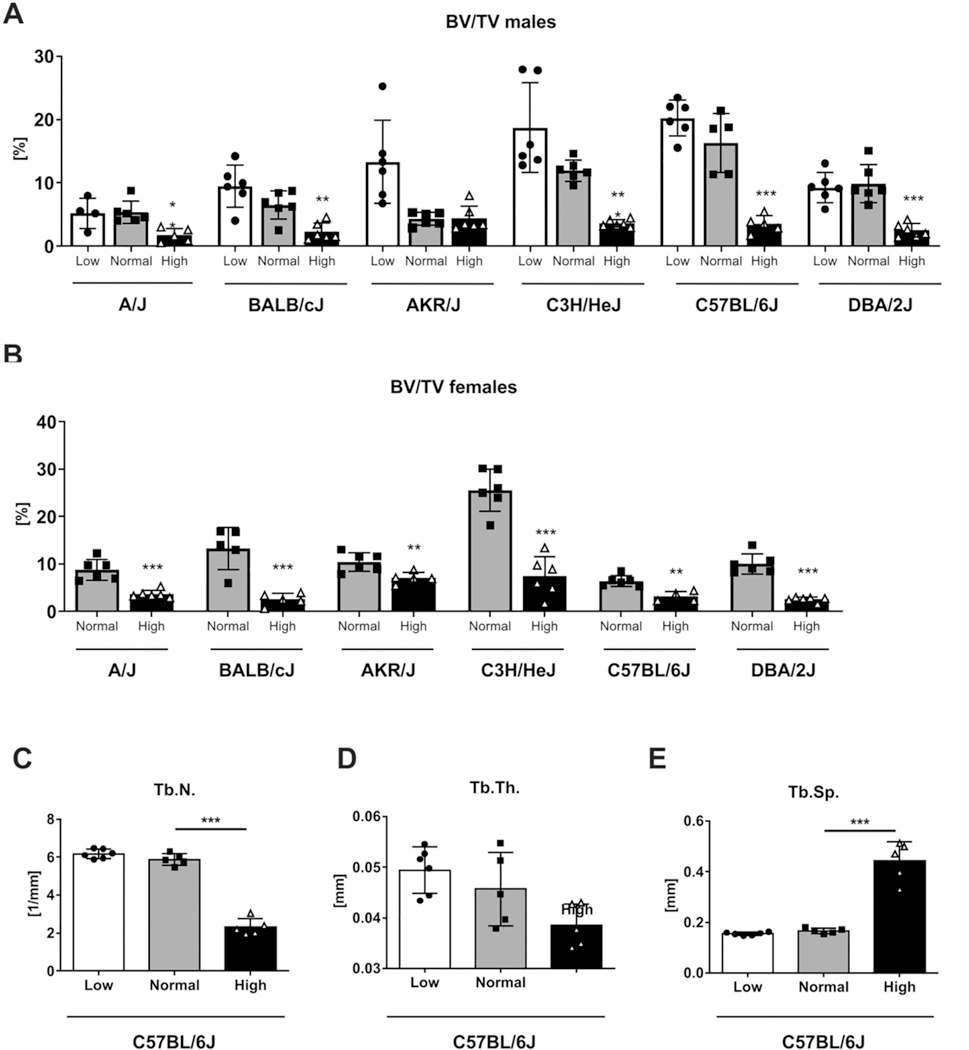
Trabecular bone microarchitecture of six different mouse strains fed different iron diets (UCLA study). Trabecular bone phenotyping of femora of 12-week old male and female mice of A/J, BALB/cJ, AKR/J, C3H/HeJ, C57BL/6J and DBA/2J mice fed low, standard, and high iron diets (UCLA cohort) using μCT measurements. Depicted are **(A**) trabecular bone volume/tissue volume (BV/TV) of male mice, **(B)** trabecular bone volume/tissue volume (BV/TV) of female mice, **(C)** trabecular number (Tb.N) of male C57BL/6J mice, **(D)** trabecular thickness (Tb.Th) of C57BL/6J male mice, and **(E)** trabecular spacing (Tb.Sp) of C57BL/6J male mice. Data represent the mean ± SD. A one-way analysis of variance (ANOVA) was used for statistical analysis. *n* = 6, ***p < 0.001 versus the UCLA iron sufficient standard diet.

**Fig. 2.**
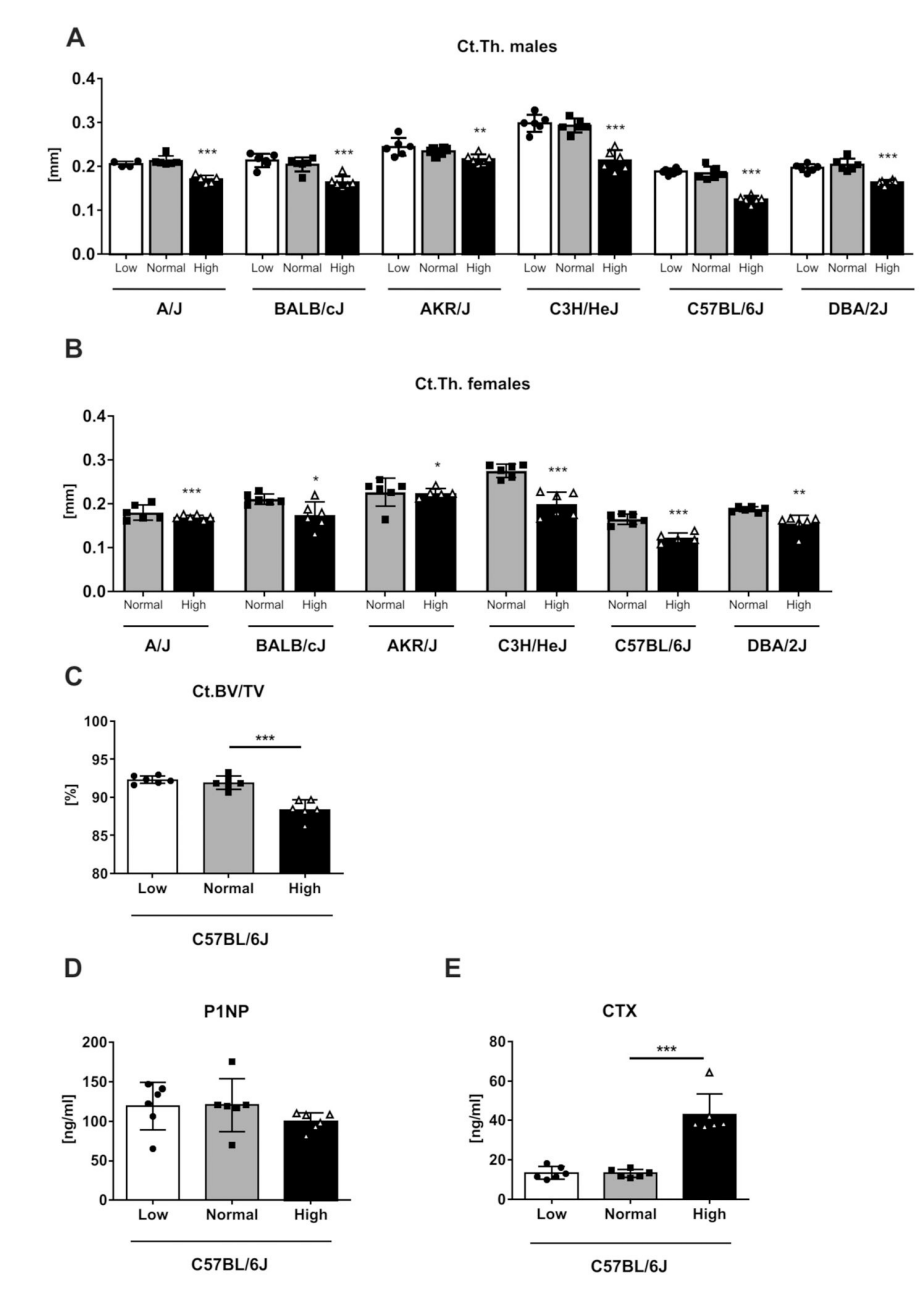
Cortical bone microarchitecture and bone turnover markers of six different mouse fed different iron diets (UCLA study). Cortical bone phenotyping of femora of 12-week old male and female mice of A/J, BALB/cJ, AKR/J, C3H/HeJ, C57BL/6J and DBA/2J mice fed low, standard, and high iron diets (UCLA cohort) using μCT measurements. Depicted are **(A**) cortical thickness (Ct.Th) of male mice, **(B)** cortical thickness (Ct.Th) of female mice, and **(C)** cortical BV/TV of male C57BL/6J mice. **(D)** Bone turnover markers procollagen type 1 amino-terminal propeptide (P1NP) and **(E)** carboxy-terminal collagen cross-links (CTX-1) were measured in the serum of male C57BL/6J mice by ELISA. Data represent the mean ± SD. A one-way analysis of variance (ANOVA) was used for statistical analysis. n = 6, ***p < 0.001 versus the UCLA iron sufficient standard diet.

On the iron sufficient standard diet, male C57BL/6J mice had the highest femoral trabecular bone volume (15 %), followed by C3H/HeJ (12 %), DBA/2J (10 %), BALB/cJ (6.5 %), A/J (5.3 %), and AKR/J (4.3 %). After 6 weeks on the high iron diet, male and female mice of nearly all inbred strains in the UCLA cohort displayed a significant reduction of trabecular bone volume in the femur, reaching a 65 % to 88 % decrease of bone mass (Table 2 and Fig. 1A). The degree of bone loss was similar in males and females in most strains (Fig. 1B). As an exception, the AKR/J strain was the only one that did not lose trabecular bone on the high iron diet in males, but they lost around 30 % bone mass in the females. C57BL/6J mice displayed the greatest trabecular bone loss at 88 % (Fig. 1A), followed by the DBA/2J, C3H/HeJ, A/J and BALB/cJ strains (Table 2). Looking at the structural level with C57BL/6J mice as a representative strain, femora had a lower trabecular number (Tb.N) and thickness (Tb.Th), whereas trabecular separation (Tb.Sp) was increased (Table 2 and Fig. 1C-E). Furthermore, the cortical bone volume and cortical thickness were uniformly decreased by the high iron diet in all strains in males and females (Table 2 and Fig. 2A-C).

To determine whether the changes in bone mass in mice fed the high iron diet are a result of decreased bone formation or increased bone resorption, we assessed bone turnover by measuring the bone resorption marker CTX and bone formation marker P1NP in the serum of the mice. The high iron diet did not change P1NP levels, but it resulted in higher levels of the bone resorption marker CTX in all strains except A/J (Table 2 and Fig. 2D+E). In AKR/J mice, CTX was increased in males on the high iron diet, even though they did not show bone loss. As opposed to other strains on the high iron diet, this strain also tended to have increased levels of P1NP on the high iron diet (Table 2). Similar to the greater accumulation of liver iron content, male mice showed more drastic increases in serum CTX levels as females, suggesting that males may be more sensitive to the high iron diet.

#### 3.1.3. Effects of iron deficiency on bone

The effects of iron deficiency on bone were only analyzed in male mice. The low iron diet had no influence on cortical bone mass in any of the six strains (Table 2 and Fig. 2A+B). Iron deficiency did not change trabecular bone microarchitecture or bone turnover in most of the strains. Only AKR/J mice showed a significantly increased trabecular bone mass (3-fold) along with a heightened P1NP response (Table 2 and Fig. 1A-E and 2D).

Overall, these data show that most mouse strains on the high iron diet showed reduced trabecular and cortical bone mass due to increased bone resorption. Moreover, iron deficiency led to a more variable bone response across the mouse strains, with some showing no (A/J, C57BL/6J, DBA/2J), mild (BALB/c, C3H/HeJ), or significant (AKR/J) bone gain.

### 3.2. Results from the TUD study

#### 3.2.1. Iron and blood parameters

In parallel to the UCLA cohort, we conducted a second iron diet study where C57BL/6J male mice were fed a high iron, a low iron, or a standard iron sufficient diet for 8 weeks, starting from weaning at an age of 4 weeks and with similar amounts of iron as used in the UCLA cohort (Suppl. Table 1). However, in contrast to the UCLA study, all TUD study diets had the same basic composition of nutrients, and only the iron content differed.

The high iron diet resulted in a 25-fold increase in liver iron content compared to mice on the standard diet (Table 3). The low iron diet however did not significantly reduce the liver iron content compared to the standard diet (Table 3). White and red blood cell counts and the number of platelets were not affected by the different iron diets (Table 3). In contrast to the UCLA C57BL/6J male cohort (Table 1 and (22)), hemoglobin and hematocrit were not significantly reduced by either the low or high iron diet. Only MCH and MCV were significant reduced in mice fed with the low iron diet and increased in mice fed the high iron diet (Table 3).

#### 3.2.2. Bone parameters

**Fig. 3.**
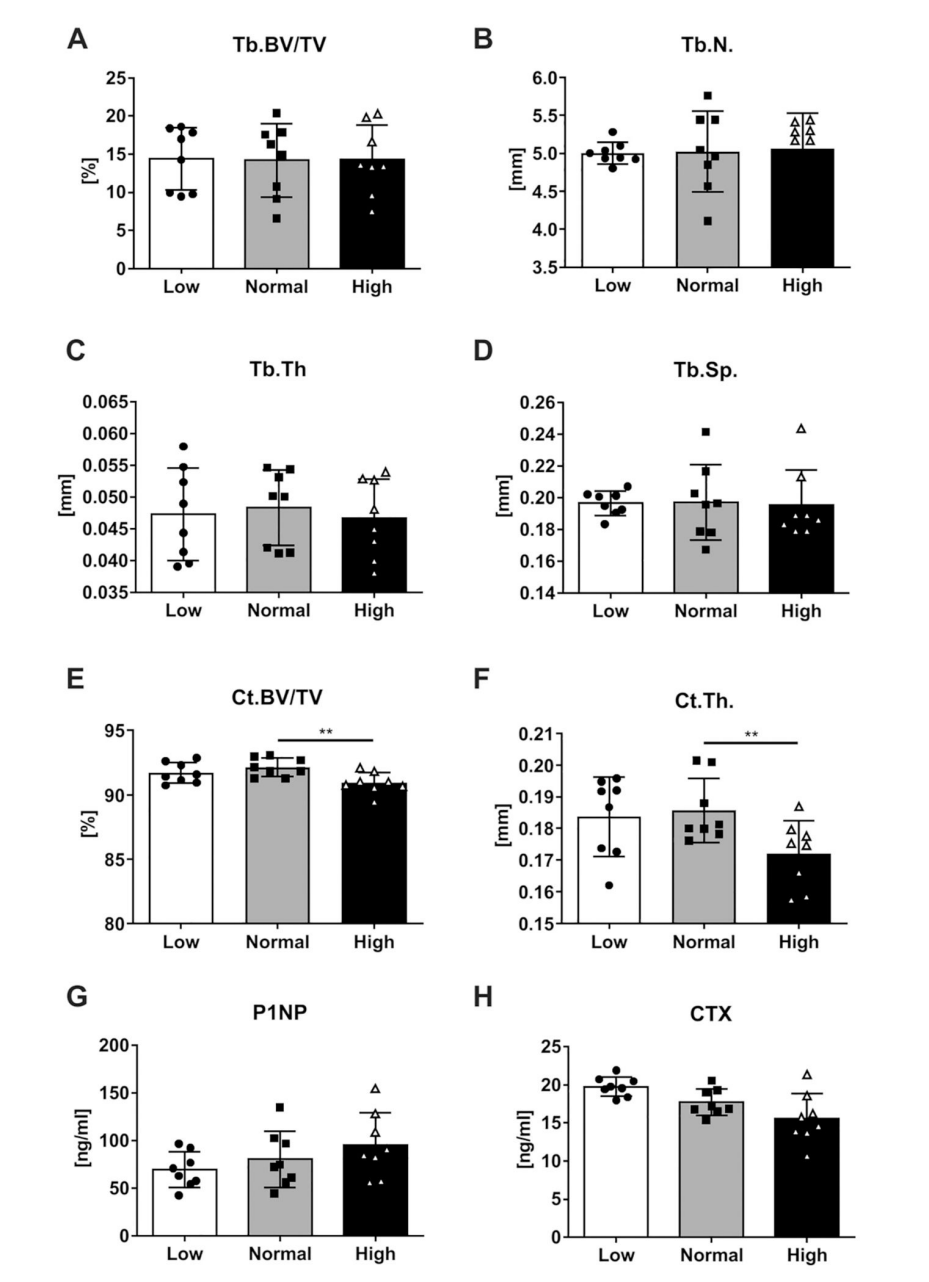
Bone microarchitecture of male C57BL/6J mice fed different iron diets (TUD study). In the TUD cohort, the high iron diet did not induce bone loss in C57BL/6J mice. Bone phenotyping was performed with μCT measurements. **(A**) Trabecular bone volume/tissue volume (BV/TV), **(B)** trabecular number (Tb.N), **(C)** trabecular thickness (Tb.Th), **(D)** trabecular spacing (Tb.Sp), **(E)** cortical BV/TV, and **(F)** cortical thickness (Ct.Th). Bone turnover markers **(G)** procollagen type 1 amino-terminal propeptide (P1NP) and **(H)** carboxy-terminal collagen cross-links (CTX-1) were measured in the serum by ELISA. Data represent the mean ± SD. A one-way analysis of variance (ANOVA) was used for statistical analysis. *n* = 8, *p < 0.05 versus the TUD iron sufficient standard diet.

Next, we investigated whether the bone was affected by the high or low iron diets in this cohort by analyzing the bone phenotype by μCT and bone turnover marker measurements. Contrarily to the UCLA cohort, trabecular bone volume (Fig. 3A) as well as trabecular structural bone parameters (Fig. 3B-D) were completely unaffected by the different TUD study iron diets. The cortical bone volume and cortical thickness were reduced however by 2–8 % in mice fed the high iron diet compared to mice fed the iron sufficient diet (Fig. 3E-F). Bone turnover markers CTX and P1NP in the serum were not affected by the diets (Fig. 3G-H).

Taken together, the data from this cohort shows that dietary iron deficiency did not impact any of the bone parameters measured, while iron overload only had negative effects on cortical, but not trabecular bone. Thus, while the effects of iron deficiency on bone were in line with the findings from the UCLA study, the impact of the high iron diets on bone differed significantly between the two studies.

### 3.3. Comparison of diet composition

Given the strong differences in high iron diet effects on bone mass between the two studies in C57BL/6J mice, we sought possible reasons. As the time of the exposure to the diets after weaning was similar, and the iron content of the diets was the same, we analyzed the diet compositions in more detail. Both high iron diets had a comparable energy density (~3720 kcal/kg TUD diet vs. ~3524 kcal/kg UCLA diet, Suppl. Table 1). However, we found that the diet used in the TUD study was much richer in minerals (~ two-fold higher concentrations of calcium, phosphorus, sodium, potassium, copper, and zinc; magnesium (3.7-fold higher), manganese (9-fold higher)) and vitamins (in particular vitamin A (4-fold higher), several B vitamins (4–8 fold higher)) (Suppl. Table 1) than the UCLA diets. Moreover, looking into detail at the UCLA diets, we noticed that the diet composition of the iron sufficient standard diet and the high/low iron diets were different. The sufficient iron diet had a lower caloric content (composition of AIN93 diets reported in [26]). Furthermore, the protein source in the UCLA low and high iron diets was derived from casein (similar to the TUD diets), while for the UCLA iron sufficient diet, individual amino acids at ratios that match what is typically found in casein were added as building blocks for proteins.

Taken together, the composition of the diet, most likely the higher amounts of minerals and vitamins in the TUD cohort diets, could account for the differences observed between the UCLA and TUD cohorts in the trabecular bone phenotype.

## 4. Discussion

Human studies show that iron overload and deficiency are both associated with lower bone strength. The underlying mechanisms, however, remain poorly understood. Rodent models of iron overload and deficiency have been used to gain more insights into the pathogenesis underlying bone loss in iron overload and iron deficiency conditions, however, with varying results. As previous studies have used various iron formations, administration routes, different mouse or rat strains, different ages and treatment duration etc., the comparability of the studies is hampered. In this study, we describe the influence of dietary iron overload and iron deficiency on bone homeostasis in six different inbred strains in male and female mice and analyzed the impact of the dietary compositions. Together, our studies suggest that dietary constituents in addition to genetic background can significant modulate the effects of both iron deficiency and iron overload on bone, which could help explain the significant variability seen in previous studies. Moreover, our study may indicate that a nutrient-rich diet could mitigate the harmful effects of iron overload on bone.

In the UCLA study, all mouse strains were responsive to the iron high and iron low diets. As expected, the iron parameters of mice fed the standard diet varied between the strains, ranging from relative high liver iron concentrations of 700–900 μg/g in AKR/J mice to low concentrations of 250–320 μg/g in C57BL/6J mice. Previous studies have established the influence of the genetic background on physiological systemic iron parameters in mice [27–32]. The relatively low iron liver content in C57BL/6J in comparison to other strains is in line with results from other studies [32]. Noteworthy, male C57BL/6J mice were most susceptible to dietary iron overload with a 30-fold increase of liver iron content on the high iron diet compared to the standard diet, whereas AKR/J mice were least susceptible to iron loading (6-fold increase). Even though the basal liver iron content in male C57BL/6J mice differed between the UCLA and TUD study (269 μg/g vs. 99 μg/g), both increased the liver iron content by 30- and 25-fold, respectively. The difference in the measured iron levels on the UCLA and TUD diets may stem in part from the different methods used to analyze liver iron content, with total liver iron measured in the UCLA cohort, while only non-heme iron was measured in the TUD cohort.

We also noted considerable differences between the two studies in the hematologic response to the different iron diets. The low iron diet induced mild anemia in all six UCLA cohort mouse strains, while in the TUD study only a decrease in mean cell volume and mean cell hemoglobin concentration reached significance. Mice fed a high iron diet also developed anemia, in particular C3H/HeJ, DBA/2J and C57BL/6J mice, as previously reported [23]. Anemia in rodents after feeding a high iron diet was reported in some [24,33,34], but not other studies, in which hemoglobin values were increased [35] or not changed at all [36]. We did not observe any relevant changes in blood parameters in the TUD C57BL/6J high iron diet cohort. The differences in response to both low and high iron diet in the two studies could be attributable to differences in the nutrient content of the diets, with the TUD diet containing more minerals and vitamins. In particular B-vitamins such as folate and vitamin B12 as well as copper have been shown to be important to maintain normal red blood cell counts [37,38].

Studies in humans have reported that conditions of iron overload as well as iron deficiency are associated with an increase in bone fragility. In both of our studies, the low iron diet had no influence on bone mass and bone turnover markers in most of the strains studied. Intriguingly, studies in rats showed that dietary iron restriction led to severely reduced hematocrit levels and reduced bone mass and strength [40–44]. Similarly, in humans, it has been demonstrated that dietary iron deficiency and iron deficiency anemia have a negative impact on bone homeostasis, especially in women [45,46]. We could not confirm any effect of the low iron diet on bone turnover in our mouse studies; however, we did not examine females on this diet. Interestingly, we only found one other study examining the impact of a low iron diet on bone in mice, which also did not show bone loss [47]. Taken together, these data suggest that mice are relatively resistant to the effects of dietary iron deficiency induced bone loss.

The most striking difference between our two cohorts was the profound trabecular bone loss in the high iron diet group of the UCLA study vs. no trabecular bone loss in the TUD study. We have two main hypotheses for the reason underlying the differences between the studies. Our first hypothesis is that differences in macronutrient content could impact the effects of iron overload. In the UCLA study, the iron sufficient standard diet contained individual amino acids instead of casein, which was used in the high iron diet. In the TUD studies, the protein source was the same for both the iron sufficient and iron overload diet. Dietary proteins have been reported to have a positive impact on calcium metabolism and bone mineral density [53]. Moreover, studies suggest that various protein sources have different effects on bone (e.g. casein vs. soy) [54]. Finally, amino acids exert various potentially even opposing effects on osteoclasts and osteoblasts. Thus, even though the individual amino acids have been added at ratios that reflect the composition of casein, it may be that the kinetics and use of amino acids is different when provided individually. To avoid confounding factors stemming from the diet, it is clear that it would have been beneficial to have the exact same background diet composition when comparing the effects of altering specific minerals, such as iron. Noteworthy, the trabecular bone mass was not different between the TUD and UCLA study groups in male C57BL/6 mice under standard iron diet. However, the basic diet compositions of the low and high iron diets in the UCLA study were the same (except for iron content), strongly suggesting that high iron was the main responsible factor for the bone loss in the high iron group.

The second hypothesis is that increased micronutrients (vitamins and mineral) in the TUD diets may have mitigated the effects of iron overload on trabecular bone. Dietary vitamin levels, of which vitamin A and B were particularly enriched in the TUD diets, have been shown to be important for proper bone health [55–58]. In addition, several minerals such as copper, manganese, zinc, and selenium are required at appropriate concentrations for maintaining bone (and overall) health, similar to the requirements for iron [59]. Particular micronutrients with anti-oxidant properties such as vitamin A as well as selenium and zinc might have been protective of trabecular bone mass loss in mice fed the high iron diet in the TUD cohort. Future studies in which the dietary composition is strictly controlled would be necessary to conclusively address this hypothesis.

Finally, another important finding of this study was the cortical bone loss in the iron overload groups of both cohorts. This finding suggests that cortical bone is more susceptible to dietary iron overload than trabecular bone. Similar findings have been reported in humans with HFE mutations, showing cortical, but not trabecular bone loss [60]. The reason for the difference is unclear, but it is possible that iron-handling factors are differentially expressed in the two bone compartments. In studies that have investigated the effects of iron overload on bone in mice, the cortical bone compartment was often neglected. Thus, these results highlight the importance of also considering the cortical bone compartment when studying the effects of iron on bone.

Besides the above-mentioned limitations of the different setups of the diets of the two studies, this study is further limited by the different treatment durations of the study (6 vs. 8 weeks), and housing in different mouse facilities, which may affect the microbiome. However, all bone parameters were measured in the same lab with the identical μCT device. This study also has several strengths, such as the inclusion of 6 different inbred strains, the inclusion of males and females, and a detailed comparison of the dietary components of the different diets used.

In summary, our data reveal that dietary iron overload has a negative influence on cortical bone parameters and highlight the need for standardized diet formulations when investigating iron overload on trabecular bone.

## Supplementary Material

Supplementary Table 1

Supplementary File

Supplementary data to this article can be found online at https://doi.org/10.1016/j.bone.2024.117092.

## Figures and Tables

**Table 1:** Blood parameters, body weight and liver iron content of C57BL/6J mice fed different iron diets in the UCLA study.

	Male			Female	
	Standard diet	Low Fe diet	High Fe diet	Standard diet	High Fe diet

C57BL/6J					
Body weight [g]	27.58 ± 3.61	24.68 ± 2.20	19.52 ± 2.50**	18.38 ± 1.94	16.44 ± 0.82
Liver Fe [μg/g]	268.9 ± 72.9	105.2 ± 18.4*	8023.5 ± 3045.2***	320.4 ± 45.5	3733.8 ± 734.4***
RBC [10^6^/μl]	10.50 ± 0.24	10.75 ± 0.39	6.26 ± 1.90***	9.56 ± 0.62	7.76 ± 1.35*
HCT [%]	44.93 ± 0.81	40.58 ± 2.31**	29.63 ± 9.02**	40.92 ± 3.43	34.98 ± 6.06
HGB [g/dl]	15.60 ± 0.37	14.17 ± 0.83**	10.22 ± 3.04**	14.20 ± 0.96	12.48 ± 2.21
MCV [fl]	42.75 ± 0.36	37.72 ± 0.88***	47.28 ± 1.52***	42.75 ± 0.91	45.08 ± 1.54*
MCH [pg]	14.83 ± 0.27	13.17 ± 0.34***	16.40 ± 0.25***	14.85 ± 0.20	16.10 ± 0.29***
MCHC [g/dl]	34.77 ± 0.71	34.95 ± 0.57	34.70 ± 0.89	34.75 ± 0.71	35.78 ± 1.05
PLT [10^3^/μl]	392.0 ± 40.8	409.7 ± 57.6	619.7 ± 234.5*	343.17 ± 100.9	423.60 ± 271.5
WBC [10^3^/μl]	7.08 ± 3.10	7.85 ± 3.63	6.60 ± 3.01	4.32 ± 3.15	4.52 ± 2.32

RBC = red blood cells, HCT = hematocrit, HGB = hemoglobin, MCV = mean corpuscular volume, MCH = mean corpuscular hemoglobin, MCHC = mean corpuscular hemoglobin concentration, PLT = platelets, WBC = white blood cells. Data represent the mean ± SD. Statistical analysis was performed by the Student’s *t*-test. For females, *N* = 6 for the standard diet and *N* = 5 for the high Fe diet. For males, N = 6 for the standard diet, *N* = 6 for the low iron diet, and N = 6 for the high Fe diet, except for liver iron, where data for one mouse on the standard diet was excluded due to extreme values for several metals. The data for the male mice fed the iron sufficient standard and high iron diets have been previously reported in another publication where bone was not studied ([24], “pilot study” results).

* p<0.05

** p<0.01

*** p<0.001

**Table 2:** Femoral trabecular and cortical bone measurements and bone turnover markers Male Female of different mouse strains fed the standard, low, and high iron diets (UCLA study).

	Male			Female	
	Standard diet	Low Fe diet	High Fe diet	Standard diet	High Fe diet

A/J	N = 6	*N* = 4	N = 5	N = 6	N = 6
Tb.BV/TV [%]	5.33 ± 1.78	5.19 ± 2.39	1.70 ± 1.07**	8.75 ± 2.21	3.61 ± 0.82***
Tb.N [1/mm]	3.36 ± 0.39	3.43 ± 0.50	1.93 ± 0.43***	3.77 ± 0.20	2.98 ± 0.19***
Tb.Th [mm]	0.042 ± 0.003	0.040 ± 0.005	0.036 ± 0.003*	0.047 ± 0.002	0.038 ± 0.002***
Tb.Sp [mm]	0.300 ± 0.034	0.296 ± 0.040	0.553 ± 0.122***	0.267 ± 0.015	0.340 ± 0.026***
Ct.BV/TV [%]	94.76 ± 0.30	94.49 ± 0.56	93.38 ± 0.46***	94.27 ± 0.52	93.30 ± 0.91*
Ct.Th [mm]	0.213 ± 0.011	0.206 ± 0.007	0.170 ± 0.009***	0.194 ± 0.010	0.169 ± 0.005***
P1NP [ng/ml]	82.57 ± 26.05	73.0 ± 20.51	67.92 ± 14.98	69.17 ± 12.81	63.35 ± 7.91
CTX [ng/ml]	16.25 ± 4.91	14.43 ± 6.39	14.58 ± 4.70	13.95 ± 3.39	32.72 ± 29.40
BALB/cJ	N = 6	*N* = 6	N = 6	N = 5	N = 5
Tb.BV/TV [%]	6.46 ± 2.23	9.47 ± 3.34	2.25 ± 1.40**	13.26 ± 4.43	2.51 ± 1.28***
Tb.N [1/mm]	3.70 ± 0.57	4.27 ± 0.76	1.88 ± 0.55***	3.91 ± 0.43	2.08 ± 0.52***
Tb.Th [mm]	0.041 ± 0.004	0.042 ± 0.004	0.040 ± 0.002	0.055 ± 0.008	0.040 ± 0.006**
Tb.Sp [mm]	0.276 ± 0.047	0.240 ± 0.057	0.581 ± 0.152***	0.260 ± 0.032	0.514 ± 0.142**
Ct.BV/TV [%]	95.01 ± 0.57	95.25 ± 0.41	93.20 ± 0.66***	94.82 ± 1.19	93.08 ± 1.17*
Ct.Th [mm]	0.204 ± 0.016	0.213 ± 0.015	0.164 ± 0.013***	0.210 ± 0.012	0.173 ± 0.030*
P1NP [ng/ml]	85.85 ± 34.15	97.29 ± 27.09	68.84 ± 34.77	62.50 ± 14.63	62.69 ± 16.25
CTX [ng/ml]	11.0 ± 5.34	12.26 ± 1.64	32.09 ± 15.03**	16.91 ± 2.38	35.28 ± 8.17***
AKR/J	N = 6	N = 6	N = 6	N = 5	N = 6
Tb.BV/TV [%]	4.28 ± 1.03	13.31 ± 6.58**	4.38 ± 1.97	10.34 ± 1.96	7.03 ± 1.15**
Tb.N [1/mm]	3.17 ± 0.27	4.06 ± 0.58**	2.50 ± 0.44**	3.62 ± 0.29	2.88 ± 0.21**
Tb.Th [mm]	0.042 ± 0.003	0.053 ± 0.008*	0.043 ± 0.006	0.053 ± 0.003	0.048 ± 0.001*
Tb.Sp [mm]	0.321 ± 0.029	0.249 ± 0.044**	0.418 ± 0.070*	0.230 ± 0.023	0.356 ± 0.026***
Ct.BV/TV [%]	95.18 ± 0.33	94.94 ± 0.81	94.48 ± 0.64*	94.79 ± 1.19	95.02 ± 0.36
Ct.Th [mm]	0.234 ± 0.008	0.244 ± 0.020	0.216 ± 0.011**	0.214 ± 0.012	0.173 ± 0.030*
P1NP [ng/ml]	76.30 ± 22.31	72.97 ± 18.61	111.62 ± 56.74	110.46 ± 22.72	113.51 ± 35.12
CTX [ng/ml]	11.13 ± 2.40	10.49 ± 2.93	22.11 ± 5.94**	23.88 ± 2.48	35.12 ± 11.83*
C3H/HeJ	N = 6	*N* = 6	N = 6	N = 6	N = 6
Tb.BV/TV [%]	11.94 ± 1.69	18.19 ± 7.77	3.57 ± 0.59***	25.53 ± 4.44	7.39 ± 4.13***
Tb.N [1/mm]	3.29 ± 0.36	3.96 ± 0.69	1.64 ± 0.13***	4.44 ± 0.30	2.45 ± 0.49***
Tb.Th [mm]	0.069 ± 0.004	0.073 ± 0.010	0.056 ± 0.006**	0.085 ± 0.007	0.056 ± 0.006***
Tb.Sp [mm]	0.309 ± 0.037	0.255 ± 0.045*	0.626 ± 0.051***	0.221 ± 0.021	0.447 ± 0.113***
Ct.BV/TV [%]	96.19 ± 0.24	95.83 ± 0.38	93.45 ± 1.19***	95.60 ± 0.21	93.53 ± 1.33**
Ct.Th [mm]	0.293 ± 0.016	0.299 ± 0.020	0.214 ± 0.023***	0.275 ± 0.015	0.198 ± 0.028***
P1NP [ng/ml]	83.88 ± 16.45	119.95 ± 10.14**	85.33 ± 23.45	104.27 ± 31.79	118.06 ± 59.90
CTX [ng/ml]	17.86 ± 5.12	20.79 ± 6.37	47.95 ± 12.95***	22.92 ± 4.26	52.03 ± 23.67*
DBA/2J	N = 6	N = 6	N = 6	N = 6	N = 6
Tb.BV/TV [%]	9.84 ± 3.03	9.25 ± 2.39	2.48 ± 1.06***	10.01 ± 2.12	2.49 ± 0.52***
Tb.N [1/mm]	4.94 ± 0.52	4.75 ± 0.32	2.13 ± 0.52***	4.36 ± 0.34	2.40 ± 0.27***
Tb.Th [mm]	0.041 ± 0.003	0.042 ± 0.003	0.038 ± 0.002	0.046 ± 0.005	0.039 ± 0.002**
Tb.Sp [mm]	0.202 ± 0.024	0.209 ± 0.014	0.498 ± 0.119***	0.259 ± 0.019	0.435 ± 0.059**
Ct.BV/TV [%]	94.13 ± 0.75	94.37 ± 0.36	92.96 ± 0.58***	94.39 ± 0.44	93.43 ± 0.28**
Ct.Th [mm]	0.204 ± 0.014	0.197 ± 0.009	0.163 ± 0.005***	0.187 ± 0.006	0.152 ± 0.022**
P1NP [ng/ml]	123.28 ± 44.93	119.44 ± 18.85	109.45 ± 36.86	345.84 ± 433.89	146.93 ± 38.47
CTX [ng/ml]	14.31 ± 3.17	18.71 ± 3.69	23.92 ± 5.49**	29.75 ± 1.18	37.50 ± 13.27

Tb.BV/TV = trabecular bone volume/total volume, Tb.N = trabecular number, Tb.Th = trabecular thickness, Tb.Sp = trabecular separation, Ct.BV/TV = cortical bone volume/total volume, Ct.Th = cortical thickness, P1NP = pro-collagen type I amino-terminal propeptide, CTX = C-terminal telopeptide. Data represent the mean ± SD. Statistical analysis was performed by the Student’s *t*-test.

* p<0.05

** p<0.01

*** p<0.001

**Table 3:** Blood parameters, body weight and liver iron content of C57BL/6J mice fed the standard, low, and high iron diets (TUD study).

	Male		
	Standard diet	Low Fe diet	High Fe diet

C57BL/6J	N = 8	N = 8	N = 8
Body weight [g]	28.25 ± 2.70	28.20 ± 2.35	27.08 ± 1.86
Liver Fe [μg/g]	98.87 ± 25.65	89.99 ± 33.06	2476.25 ± 973.88***
RBC [10^6^/μl]	9.88 ± 0.92	9.95 ± 0.80	9.38 ± 0.83
HCT [%]	50.2 ± 5.0	49.2 ± 4.8	51.5 ± 4.4
HGB [g/dl]	9.11 ± 0.83	9.00 ± 0.73	9.41 ± 0.77
MCV [fl]	50.80 ± 0.78	49.43 ± 1.11*	54.94 ± 1.01***
MCH [pg]	0.922 ± 0.007	0.905 ± 0.018*	1.004 ± 0.013***
MCHC [g/dl]	18.18 ± 0.24	18.30 ± 0.48	18.28 ± 0.32
PLT [10^3^/μl]	688.9 ± 97.5	642.4 ± 243.7	851.3 ± 278.8
WBC [10^3^/μl]	8.34 ± 3.11	8.23 ± 2.26	10.40 ± 2.40

RBC = red blood cells, HCT = hematocrit, HGB = hemoglobin, MCV = mean corpuscular volume, MCH = mean corpuscular hemoglobin, MCHC = mean corpuscular hemoglobin concentration, PLT = platelets, WBC = white blood cells. Data represent the mean ± SD. Statistical analysis was performed by the Student’s *t*-test.

* p<0.05

** p<0.01

*** p<0.001

## Data Availability

Data will be made available on request.

## References

[1] Camaschella C, Iron-deficiency anemia N Engl. J. Med. 373 (5) (2015) 485–486, 10.1056/NEJMc1507104. 26222573

[2] Brissot P, Pietrangelo A, Adams PC, de Graaff B, McLaren CE, Loreal O, Haemochromatosis, Nat. Rev. Dis. Primers 4 (2018) 18016, 10.1038/nrdp.2018.16. 29620054 PMC7775623

[3] Roth MP, Meynard D, Coppin H, Regulators of hepcidin expression, Vitam. Horm. 110 (2019) 101–129, 10.1016/bs.vh.2019.01.005. 30798807

[4] Nemeth E, Tuttle MS, Powelson J, Vaughn MB, Donovan A, Ward DM, Ganz T, Kaplan J, Hepcidin regulates cellular iron efflux by binding to ferroportin and inducing its internalization, Science 306 (5704) (2004) 2090–2093, 10.1126/science.1104742. 15514116

[5] Pilling LC, Tamosauskaite J, Jones G, Wood AR, Jones L, Kuo CL, Kuchel GA, Ferrucci L, Melzer D, Common conditions associated with hereditary haemochromatosis genetic variants: cohort study in UK Biobank, BMJ 364 (2019) k5222, 10.1136/bmj.k5222. 30651232 PMC6334179

[6] Ledesma-Colunga MG, Weidner H, Vujic Spasic M, Hofbauer LC, Baschant U, Rauner M, Shaping the bone through iron and iron-related proteins, Semin. Hematol. 58 (3) (2021) 188–200, 10.1053/j.seminhematol.2021.06.002. 34389111

[7] Ishii KA, Fumoto T, Iwai K, Takeshita S, Ito M, Shimohata N, Aburatani H, Taketani S, Lelliott CJ, Vidal-Puig A, Ikeda K, Coordination of PGC-1beta and iron uptake in mitochondrial biogenesis and osteoclast activation, Nat. Med. 15 (3) (2009) 259–266, 10.1038/nm.1910. 19252502

[8] Baschant U, Rauner M, Balaian E, Weidner H, Roetto A, Platzbecker U, Hofbauer LC, Wnt5a is a key target for the pro-osteogenic effects of iron chelation on osteoblast progenitors, Haematologica 101 (12) (2016) 1499–1507, 10.3324/haematol.2016.144808. 27540134 PMC5479606

[9] Jing X, Du T, Chen K, Guo J, Xiang W, Yao X, Sun K, Ye Y, Guo F, Icariin protects against iron overload-induced bone loss via suppressing oxidative stress, J. Cell. Physiol. 234 (7) (2019) 10123–10137, 10.1002/jcp.27678. 30387158

[10] Doyard M, Chappard D, Leroyer P, Roth MP, Loreal O, Guggenbuhl P, Decreased bone formation explains osteoporosis in a genetic mouse model of Hemochromatosiss, PLoS One 11 (2) (2016) e0148292, 10.1371/journal.pone.0148292. PMC4734777 26829642

[11] Guggenbuhl P, Deugnier Y, Boisdet JF, Rolland Y, Perdriger A, Pawlotsky Y, Chales G, Bone mineral density in men with genetic hemochromatosis and HFE gene mutation, Osteoporos. Int. 16 (12) (2005) 1809–1814. 15928800 10.1007/s00198-005-1934-0

[12] Valenti L, Varenna M, Fracanzani AL, Rossi V, Fargion S, Sinigaglia L, Association between iron overload and osteoporosis in patients with hereditary hemochromatosis, Osteoporos. Int. 20 (4) (2009) 549–555, 10.1007/s00198-008-0701-4. 18661088

[13] Burden A, Burkhard T, Rauner M, Immoos M, Hofbauer LC, Steinbicker AU, Increased fracture risk associated with iron overload: a population-based matched cohort study, J. Bone Miner. Res. 38 (S1) (2022) 1–364.36461761

[14] Kim BJ, Lee SH, Koh JM, Kim GS, The association between higher serum ferritin level and lower bone mineral density is prominent in women >/=45 years of age (KNHANES 2008–2010), Osteoporos. Int. 24 (10) (2013) 2627–2637, 10.1007/s00198-013-2363-0. 23592044

[15] Peng P, Xiao F, Gao S, Fang W, Lin T, He W, Wei Q, Association between serum ferritin and bone mineral density in US adults, J. Orthop. Surg. Res. 17 (1) (2022) 494, 10.1186/s13018-022-03357-1. 36384633 PMC9670566

[16] Lu M, Liu Y, Shao M, Tesfaye GC, Yang S, Associations of Iron intake, serum Iron and serum ferritin with bone mineral density in women: the National Health and nutrition examination survey, 2005–2010, Calcif. Tissue Int. 106 (3) (2020) 232–238, 10.1007/s00223-019-00627-9. 31754762

[17] Tsay J, Yang Z, Ross FP, Cunningham-Rundles S, Lin H, Coleman R, Mayer-Kuckuk P, Doty SB, Grady RW, Giardina PJ, Boskey AL, Vogiatzi MG, Bone loss caused by iron overload in a murine model: importance of oxidative stress, Blood 116 (14) (2010) 2582–2589, 10.1182/blood-2009-12-260083. 20554970 PMC2953890

[18] Simao M, Camacho A, Ostertag A, Cohen-Solal M, Pinto IJ, Porto G, Hang Korng E, Cancela ML, Iron-enriched diet contributes to early onset of osteoporotic phenotype in a mouse model of hereditary hemochromatosis, PLoS One 13 (11) (2018) e0207441, 10.1371/journal.pone.0207441. PMC6241130 30427936

[19] Guggenbuhl P, Fergelot P, Doyard M, Libouban H, Roth MP, Gallois Y, Chales G, Loreal O, Chappard D, Bone status in a mouse model of genetic hemochromatosis, Osteoporos. Int. 22 (8) (2011) 2313–2319, 10.1007/s00198-010-1456-2. 20976594

[20] Ledesma-Colunga MG, Baschant U, Fiedler IAK, Busse B, Hofbauer LC, Muckenthaler MU, Altamura S, Rauner M, Disruption of the hepcidin/ferroportin regulatory circuitry causes low axial bone mass in mice, Bone 137 (2020) 115400, 10.1016/j.bone.2020.115400. 32380257

[21] Wagner A, Alan B, Yilmaz D, Ahmad M, Liu P, Tangudu NK, Tuckermann JP, Vujic Spasic M, Despite genetic Iron overload, Hfe-hemochromatosis mice do not show bone loss, JBMR Plus 3 (9) (2019) e10206, 10.1002/jbm4.10206. PMC6808227 31667458

[22] Percie du Sert N, Hurst V, Ahluwalia A, Alam S, Avey MT, Baker M, Browne WJ, Clark A, Cuthill IC, Dirnagl U, Emerson M, Garner P, Holgate ST, Howells DW, Karp NA, Lazic SE, Lidster K, MacCallum CJ, Macleod M, Pearl EJ, Petersen OH, Rawle F, Reynolds P, Rooney K, Sena ES, Silberberg SD, Steckler T, Wurbel H, The ARRIVE guidelines 2.0: Updated guidelines for reporting animal research, PLoS Biol. 18 (7) (2020) e3000410, 10.1371/journal.pbio.3000410. PMC7360023 32663219

[23] McLachlan S, Page KE, Lee SM, Loguinov A, Valore E, Hui ST, Jung G, Zhou J, Lusis AJ, Fuqua B, Ganz T, Nemeth E, Vulpe CD, Hamp1 mRNA and plasma hepcidin levels are influenced by sex and strain but do not predict tissue iron levels in inbred mice, Am. J. Physiol. Gastrointest. Liver Physiol. 313 (5) (2017) G511–G523, 10.1152/ajpgi.00307.2016. 28798083 PMC5792216

[24] Brie K Fuqua LM, Stela McLachlan, Calvin Pan, Davis Richard C., Simon T. hui, Nam Che, Zhiqiang Zhou, Carmen Ng, Charugundla Sarada, Blencowe Montgomery, Saleem Zara, Miikeda Aika, Ozdemir Beyza, Hui Chester, Li Thy, Stolin Clara L., Kozuch Marianne, Zhou Jie, Page Kathryn, Irimagawa Hiro, Ku Nam, Taraszka Kodi, LaPierre Nathan, Killilea David W., Frazer David M., Yang Xia, Eskin Eleazar, Vulpe Chris D., Lusis Aldons J. (2023) The Genetic Architecture of Dietary Iron Overload and Associated Pathology in Mice. bioRxiv. doi:10.1101/2023.06.05.543764.

[25] Dempster DW, Compston JE, Drezner MK, Glorieux FH, Kanis JA, Malluche H, Meunier PJ, Ott SM, Recker RR, Parfitt AM, Standardized nomenclature, symbols, and units for bone histomorphometry: a 2012 update of the report of the ASBMR Histomorphometry nomenclature committee, J. Bone Miner. Res. 28 (1) (2013) 2–17, 10.1002/jbmr.1805. 23197339 PMC3672237

[26] Reeves PG, Nielsen FH, Fahey GC Jr., AIN-93 purified diets for laboratory rodents: final report of the American Institute of Nutrition ad hoc writing committee on the reformulation of the AIN-76A rodent diet, J. Nutr. 123 (11) (1993) 1939–1951, 10.1093/jn/123.11.1939. 8229312

[27] Champy MF, Selloum M, Zeitler V, Caradec C, Jung B, Rousseau S, Pouilly L, Sorg T, Auwerx J, Genetic background determines metabolic phenotypes in the mouse, Mamm. Genome 19 (5) (2008) 318–331, 10.1007/s00335-008-9107-z. 18392653

[28] Courselaud B, Troadec MB, Fruchon S, Ilyin G, Borot N, Leroyer P, Coppin H, Brissot P, Roth MP, Loreal O, Strain and gender modulate hepatic hepcidin 1 and 2 mRNA expression in mice, Blood Cells Mol. Dis. 32 (2) (2004) 283–289, 10.1016/j.bcmd.2003.11.003. 15003819

[29] Dupic F, Fruchon S, Bensaid M, Borot N, Radosavljevic M, Loreal O, Brissot P, Gilfillan S, Bahram S, Coppin H, Roth MP, Inactivation of the hemochromatosis gene differentially regulates duodenal expression of iron-related mRNAs between mouse strains, Gastroenterology 122 (3) (2002) 745–751, 10.1053/gast.2002.31877. 11875007

[30] Leboeuf RC, Tolson D, Heinecke JW, Dissociation between tissue iron concentrations and transferrin saturation among inbred mouse strains, J. Lab. Clin. Med. 126 (2) (1995) 128–136. 7636385

[31] Wang F, Paradkar PN, Custodio AO, McVey Ward D, Fleming MD, Campagna D, Roberts KA, Boyartchuk V, Dietrich WF, Kaplan J, Andrews NC, Genetic variation in Mon1a affects protein trafficking and modifies macrophage iron loading in mice, Nat. Genet. 39 (8) (2007) 1025–1032, 10.1038/ng2059. 17632513

[32] Cavey T, Ropert M, de Tayrac M, Bardou-Jacquet E, Island ML, Leroyer P, Bendavid C, Brissot P, Loreal O, Mouse genetic background impacts both on iron and non-iron metals parameters and on their relationships, Biometals 28 (4) (2015) 733–743, 10.1007/s10534-015-9862-8. 26041486

[33] Ha JH, Doguer C, Wang X, Flores SR, Collins JF, High-Iron consumption impairs growth and causes copper-deficiency anemia in weanling Sprague-Dawley rats, PLoS One 11 (8) (2016) e0161033, 10.1371/journal.pone.0161033. PMC4990348 27537180

[34] Wang T, Xiang P, Ha JH, Wang X, Doguer C, Flores SRL, Kang YJ, Collins JF, Copper supplementation reverses dietary iron overload-induced pathologies in mice, J. Nutr. Biochem. 59 (2018) 56–63, 10.1016/j.jnutbio.2018.05.006. 29960117 PMC6467079

[35] Nam H, Knutson MD, Effect of dietary iron deficiency and overload on the expression of ZIP metal-ion transporters in rat liver, Biometals 25 (1) (2012) 115–124, 10.1007/s10534-011-9487-5. 21826460 PMC4598638

[36] Yu F, Hao S, Yang B, Zhao Y, Zhang R, Zhang W, Yang J, Chen J, Insulin resistance due to dietary iron overload disrupts inner hair cell ribbon synapse plasticity in male mice, Neurosci. Lett. 597 (2015) 183–188, 10.1016/j.neulet.2015.04.049. 25956034

[37] Koury MJ, Ponka P, New insights into erythropoiesis: the roles of folate, vitamin B12, and iron, Annu. Rev. Nutr. 24 (2004) 105–131, 10.1146/annurev.nutr.24.012003.132306. 15189115

[38] Ha JH, Doguer C, Collins JF, Consumption of a high-Iron diet disrupts homeostatic regulation of intestinal copper absorption in adolescent mice, Am. J. Physiol. Gastrointest. Liver Physiol. 313 (4) (2017) G360–G535, 10.1152/ajpgi.00169.2017. PMC5668571 28619730

[39] referance

[40] Medeiros DM, Plattner A, Jennings D, Stoecker B, Bone morphology, strength and density are compromised in iron-deficient rats and exacerbated by calcium restriction, J. Nutr. 132 (10) (2002) 3135–3141, 10.1093/jn/131.10.3135. 12368407

[41] Medeiros DM, Stoecker B, Plattner A, Jennings D, Haub M, Iron deficiency negatively affects vertebrae and femurs of rats independently of energy intake and body weight, J. Nutr. 134 (11) (2004) 3061–3067, 10.1093/jn/134.11.3061. 15514276

[42] Diaz-Castro J, Lopez-Frias MR, Campos MS, Lopez-Frias M, Alferez MJ, Nestares T, Ojeda ML, Lopez-Aliaga I, Severe nutritional iron-deficiency anaemia has a negative effect on some bone turnover biomarkers in rats, Eur. J. Nutr. 51 (2) (2012) 241–247, 10.1007/s00394-011-0212-5. 21647667

[43] Katsumata S, Katsumata-Tsuboi R, Uehara M, Suzuki K, Severe iron deficiency decreases both bone formation and bone resorption in rats, J. Nutr. 139 (2) (2009) 238–243, 10.3945/jn.108.093757. 19106323

[44] Katsumata S, Tsuboi R, Uehara M, Suzuki K, Dietary iron deficiency decreases serum osteocalcin concentration and bone mineral density in rats, Biosci. Biotechnol. Biochem. 70 (10) (2006) 2547–2550, 10.1271/bbb.60221. 17031035

[45] Hannemann A, Nauck M, Volzke H, Weidner H, Platzbecker U, Hofbauer LC, Rauner M, Baschant U, Interactions of Anemia, FGF-23, and bone in healthy adults-results from the study of health in Pomerania (SHIP), J. Clin. Endocrinol. Metab. 106 (1) (2021) e288–e299, 10.1210/clinem/dgaa716. 33034626

[46] Harris MM, Houtkooper LB, Stanford VA, Parkhill C, Weber JL, Flint-Wagner H, Weiss L, Going SB, Lohman TG, Dietary iron is associated with bone mineral density in healthy postmenopausal women, J. Nutr. 133 (11) (2003) 3598–3602, 10.1093/jn/133.11.3598. 14608080

[47] Malecki EA, Buhl KM, Beard JL, Jacobs CR, Connor JR, Donahue HJ, Bone structural and mechanical properties are affected by hypotransferrinemia but not by iron deficiency in mice, J. Bone Miner. Res. 15 (2) (2000) 271–277, 10.1359/jbmr.2000.15.2.271. 10703928

[53] Hannan MT, Tucker KL, Dawson-Hughes B, Cupples LA, Felson DT, Kiel DP, Effect of dietary protein on bone loss in elderly men and women: the Framingham osteoporosis study, J. Bone Miner. Res. 12 (2000) 2504–2512, 10.1359/jbmr.2000.15.12.2504. 11127216

[54] Hoppe C, Molgaard C, Juul A, Michaelsen KF, High intakes of skimmed milk, but not meat, increase serum IGF-I and IGFBP-3 in eight-year-old boys, Eur. J. Clin. Nutr. 58 (9) (2004) 1211–1216, 10.1038/sj.ejcn.1601948. 15054433

[55] Singh P, Telnova S, Zhou B, Mohamed AD, Mello V, Wackerhage H, Guo XE, Panda AK, Yadav VK, Maternal vitamin B_12_ in mice positively regulates bone, but not muscle mass and strength in post-weaning and mature offspring, Am. J. Phys. Regul. Integr. Comp. Phys. 320 (6) (2021) R984–R993, 10.1152/ajpregu.00355.2020. PMC8285619 33759575

[56] Khojah Q, AlRumaihi S, AlRajeh G, Aburas A, AlOthman A, Ferwana M, Vitamin a and its dervatives effect on bone mineral density, a systematic review, J. Family Med. Prim. Care 10 (11) (2021) 4089–4095, 10.4103/jfmpc. 35136772 PMC8797105

[57] Clements M, Heffernan M, Ward M, Hoey L, Doherty LC, Hack Mendes R, Clarke MM, Hughes CF, Love I, Murphy S, McDermott E, Grehan J, McCann A, McAnena LB, Strain JJ, Brennan L, McNulty H, A 2-year randomized controlled trial with low-dose B-vitamin supplementation shows benefits on bone mineral density in adults with lower B12 status, J. Bone Miner. Res. 37 (12) (2022) 2443–2455, 10.1002/jbmr.4709. 36128889 PMC10092614

[58] Singh P, Telnova S, Zhou B, Mohamed AD, Mello V, Wackerhage H, Guo XE, Panda AK, Yadav VK, Maternal vitamin B(12) in mice positively regulates bone, but not muscle mass and strength in post-weaning and mature offspring, Am. J. Phys. Regul. Integr. Comp. Phys. 320 (6) (2021) R984–R993, 10.1152/ajpregu.00355.2020. PMC8285619 33759575

[59] Dos Santos TS, Augusto KVZ, Han Y, Sartori MMP, Batistioli JS, Contin Neto AC, Ferreira Netto RG, Zanetti LH, Pasquali GAM, Muro EM, Araujo RGAC, Basso RM, Guimaraes VY, Takahira RK, Kim WK, Sartori JR, ˜ Effects of dietary copper and zinc hydroxychloride supplementation on bone development, skin quality and hematological parameters of broilers chickens, J Anim Physiol Anim Nutr (Berl). 5 (2023) 1241–1250, 10.1111/jpn.13829. 37158583

[60] Jandl NM, Rolvien T, Schmidt T, Mussawy H, Nielsen P, Oheim R, Amling M, Barvencik F, Impaired bone microarchitecture in patients with hereditary hemochromatosis and skeletal complications, Calcif. Tissue Int. 106 (5) (2020) 465–475, 10.1007/s00223-020-00658-7. 31989186

