# Supplementary material for: Effects of dietary iron deficiency or overload on bone: Dietary details matter
Source: Bone. Author manuscript; Available in PMC 2026 Aug 10. (PMC13456107; doi:10.1016/j.bone.2024.117092)
Supplement: Supplementary file 1 [file NIHMS2184568-supplement-Supplementary_Table_1.docx]

**Suppl.Table 1. Diet composition** - Ingredients and estimated final mineral and vitamin compositions of the study diets fed in the TUD and UCLA studies. The UCLA study diets are based on the AIN93 diets reported by Reeves et al. (reference 25).

|  | ***High iron diets*** | | | ***Low iron diets*** | | | ***Sufficient iron standard diets*** | | | |
| --- | --- | --- | --- | --- | --- | --- | --- | --- | --- | --- |
|  | TUD | | UCLA | TUD | UCLA | | TUD | UCLA | | |
| **Ingredients** | % diet by weight | | | % diet by weight | | | % diet by weight | | | |
| Casein or purified L-amino acids | 20 | | 14 | 20 | 14 | | 20 | 17.65 | | |
| Sucrose* | 10 | | 8 | 10 | 10 | | 10 | 10 | | |
| Cornstarch | 40 | | 46.57 | 40 | 46.57 | | 40 | 39.97 | | |
| Dyetrose | 0 | | 15.5 | 0 | 15.5 | | 0 | 14.5 | | |
| Maltodextrin | 12.1 | | 0 | 14.1 | 0 | | 14 | 0 | | |
| L-Cystine | 0.2 | | 0.18 | 0.2 | 0.18 | | 0.2 | 0.37 | | |
| Cellulose | 3 | | 5 | 3 | 5 | | 3 | 5 | | |
| Soybean Oil | 5.5 | | 4 | 5.5 | 4 | | 5.5 | 7 | | |
| t-Butylhydroquinone | 0 | | 0.0008 | 0 | 0.0008 | | 0 | 0.0014 | | |
| Mineral mix (no iron) | 6 | | 3.5 | 6 | 3.5 | | 6 | 3.5 | | |
| Vitamin mix | 1 | | 1 | 1 | 1 | | 1 | 1 | | |
| Choline Bitartrate | 0 | | 0.25 | 0 | 0.25 | | 0 | 0.25 | | |
| Choline chloride | 0.2 | | 0 | 0.2 | 0 | | 0.2 | 0 | | |
| Carbonyl iron | 2 | | 2 | 0 | 0 | | 0 | 0 | | |
| Ferric citrate | 0 | | 0 | 0 | 0 | | 0 | 0.021 | | |
| Fe fumarate/citrate/sulfate mix | 0 | | 0 | 0 | 0 | | 0.08 | 0 | | |
| Sodium bicarbonate | 0 | | 0 | 0 | 0 | | 0 | 0.74 | | |
| Coloring | 0 | | 0 | 0 | 0 | | 0.02 | 0 | | |
| **TOTAL** | **100** | | **100** | **100** | **100** | | **100** | **100** | | |
| **Estimated kcal/kg** | **3720** | | **3524** | **3800** | **3604** | | **3796** | **3804** | | |
|  |  | |  |  |  | |  |  | | |
| **Final mineral composition** (estimated) | mg/kg diet | | | mg/kg diet | | | mg/kg diet | | | |
| Calcium | 9,300 | 5,000 | | 9,300 | | 5,000 | 9,300 | | 5,000 | |
| Phosphorus | 6,500 | 3,000 | | 6,500 | | 3,000 | 6,500 | | 3,000 | |
| Sodium | 2,000 | 1,033 | | 2,000 | | 1,033 | 2,000 | | 1,039 | |
| Potassium | 9,600 | 3,600 | | 9,600 | | 3,600 | 9,600 | | 3,600 | |
| Selenium | 0.2 | 0.17 | | 0.2 | | 0.17 | 0.2 | | 0.18 | |
| Zinc | 68 | 35 | | 68 | | 35 | 68 | | 38 | |
| Iron | 20,000 | 20,000 | | 10 | | 12 | 168 | | 45 | |
| Copper | 13 | 6 | | 13 | | 6 | 13 | | 6 | |
| Magnesium | 1,900 | 511 | | 1,900 | | 511 | 1,900 | | 513 | |
| Manganese | 98 | 10 | | 98 | | 10 | 98 | | 10 | |
|  |  |  | |  | |  |  | | |  |
| **Final vitamin composition**  (estimated) | units/kg diet | | | units/kg diet | | | units/kg diet | | | |
| Niacin (mg) | 49 | 30 | | 49 | | 30 | 49 | | 30 | |
| Calcium pantothenate (mg) | 0 | 15 | | 0 | | 15 | 0 | | 15 | |
| Pantothenic acid (mg) | 55 | 0 | | 55 | | 0 | 55 | | 0 | |
| Pyridoxine (mg) | 16 | 6 | | 16 | | 6 | 16 | | 6 | |
| Thiamine (mg) | 26 | 5 | | 26 | | 5 | 26 | | 5 | |
| Riboflavin (mg) | 16 | 6 | | 16 | | 6 | 16 | | 6 | |
| Folic acid (mg) | 16 | 2 | | 16 | | 2 | 16 | | 2 | |
| Biotin (mg) | 0.3 | 0.2 | | 0.3 | | 0.2 | 0.3 | | 0.2 | |
| Vitamin E Acetate (mg) | 150 | 150 | | 150 | | 150 | 150 | | 150 | |
| Vitamin B12 (µg) | 30 | 25 | | 30 | | 25 | 30 | | 25 | |
| Vitamin A (IU) | 15,000 | 4,000 | | 15,000 | | 4,000 | 15,000 | | 4,000 | |
| Vitamin D3 (IU) | 1,500 | 1,000 | | 1,500 | | 1,000 | 1,500 | | 1,000 | |
| Vitamin K1 (phylloquinone, mg) | 0 | 0.86 | | 0 | | 0.86 | 0 | | 0.9 | |
| Vitamin K3 (menadione nicotinamide bisulfite, mg) | 20 | 0 | | 20 | | 0 | 20 | | 0 | |
| Choline (mg) | 920 | 1,000 | | 920 | | 1,000 | 920 | | 1,000 | |
